# Modeling of Polymer Composites and Nanocomposites (2nd Edition)

**DOI:** 10.3390/polym18192385

**Published:** 2026-09-30

**Authors:** Rafał Grzejda

**Affiliations:** Faculty of Mechanical Engineering and Mechatronics, West Pomeranian University of Technology in Szczecin, 19 Piastow Ave., 70-310 Szczecin, Poland; rafal.grzejda@zut.edu.pl

## 1. Introduction

Polymer composites (PCs) and nanocomposites (PNs) have emerged as key materials in modern engineering, offering superior mechanical, thermal, electrical, and functional properties compared to traditional polymers. The inclusion of nanoscale fillers—such as carbon nanotubes, graphene, nanoclays, and cellulose nanofibers—enables unprecedented improvements in strength, durability, conductivity, and multifunctionality, driving their adoption across the aerospace, automotive, biomedical, and environmental sectors [1,2,3].

The complexity of these materials stems from their hierarchical structure, in which interactions at the nanoscale and interfacial phenomena have a decisive influence on macroscopic properties. Accurate modeling is essential for predicting, optimizing, and tailoring these properties to specific applications. Traditional analytical and numerical methods, while serving as a foundation, are often unable to capture the complex behavior of PNs, which requires the use of advanced multiscale, hybrid, and data-driven approaches [4,5,6].

In recent years, there has been a rapid rise in the popularity of combining machine learning (ML) and physics-based models with traditional modeling, enabling rapid property prediction, virtual screening, and accelerated materials design. These advances are not only transforming computational materials science but also bridging the gap between theory and industrial practice [7,8].

Some overview of the latest research findings on PCs and PNs is available in the Special Issue of Polymers entitled “Modeling of Polymer Composites and Nanocomposites” (see [9]). An extension of this overview is presented in the Special Issue of Polymers called “Modeling of Polymer Composites and Nanocomposites (2nd Edition)”, the introduction to which is this Editorial.

## 2. Overview of Papers Published in the Special Issue

Finding eco-friendly alternatives to synthetic materials used in construction for sound insulation is essential for ensuring environmental sustainability. Biocomposites (BCs) made from natural fibers combined with a biopolymer matrix appear to be a promising solution in this area. Alba et al. (Contribution No. 1) prepared soy protein BCs containing 10, 15, and 20 wt.% sheep’s wool and then incorporated spent coffee grounds through freeze-drying to obtain fibrous-porous BCs for acoustic applications [10]. Transmission loss measurements for BCs demonstrated good insulating properties, with maximum values of around 22 dB at 2500 Hz and even better performance than that of commercial synthetic solutions. The sound absorption coefficients obtained were competitive, reaching a value close to unity at mid and high frequencies. Airflow resistance [11] was determined, and the values were higher for BCs with coffee grounds, specifically 14–18 kPa·s·m^−2^ compared to 5.62–11.6 kPa·s·m^−2^. Using the measured air flow resistance data, an empirical model was developed using a genetic algorithm as a tool for predicting the sound absorption properties of the samples. This revealed the potential of the tested BCs as competitive sound insulators and absorbers in construction.

Although the use of inositol in the plastics industry still requires further research and optimization of production processes, its potential as a component of biopolymers, additives to synthetic materials, and functional coatings makes it a valuable compound in modern materials science. An example of the use of myo-inositol as an additive to rigid polyurethane/polyisocyanurate foams [12,13] is shown in Liszkowska and Moraczewski (Contribution No. 2). They examined the effect of this additive on the degradation and performance properties of the foam, as well as its structure. The parameters tested included production temperature, processing time, strength, absorbency, and flammability. The foams were aged in a special degradation chamber. The addition of myo-inositol resulted in a reduction in the diameter of the foam cells (measured in both directions). Water absorption and retention decreased. The compressive strength of the foam increased, and its flammability decreased (i.e., retention increased and the burning rate decreased).

The wear resistance of PCs depends on several factors, including the type of reinforcement, the amount of filler, the applied stress, the sliding speed and the sliding distance. Synthetic fibers, such as glass fibers, increase wear resistance due to their high strength and surface hardness [14,15]. On the other hand, natural fibers, such as those made from bamboo or kenaf, are gaining popularity due to their low cost, satisfactory mechanical properties and environmental friendliness associated with biodegradation processes [16,17,18]. With this in mind, Ahmad et al. (Contribution No. 3) provided a comparison of composites reinforced with nanoclay from fiberglass and epoxy resin and composites reinforced with bamboo fiber and epoxy resin, using Response Surface Methodology (RSM) and Artificial Neural Networks (ANNs) to predict wear resistance under the influence of various factors at different levels. The addition of nanoclay significantly improves the wear performance of both types of composites. This is primarily due to the increased hardness of the matrix and improved interlayer bonding between the fibers and the epoxy matrix. Scanning Electron Microscopy (SEM) analyses showed improvements characterized by smooth worn surfaces, reduced cracking and reduced fiber pull-out. Of the two composites, the glass fiber composite has higher overall wear resistance. However, the bamboo fiber composite shows a significant increase in wear resistance when nanoclay is added.

Lattice structures (LSs) are typically characterized by repetitive patterns of one or more geometric cells arranged within the volume of a given object [19,20,21]. Their application is aimed at reducing the weight of the structure while maintaining the external shape of the geometry and generally improving the mechanical properties [22]. An analysis of LSs printed from EPAX resin [23] used in the production of motorcycle throttle cams using RSM and ANNs was presented by Donnici et al. (Contribution No. 4). They confirmed in their research that both approaches are correct for the analysis of the parameterized core and for the identification of optimal and suboptimal design points. RSM and ANNs models can predict with high accuracy the design relationships between the saved mass and the measured displacement in the throttle cam limit switch. The proposed approach was considered suitable as a reference guide for the design of core lattices, allowing uncontrolled applications to be avoided.

LSs can be created using so-called triply periodic minimal surfaces [24,25]. These surfaces consist of repeating patterns without self-intersections and have zero mean curvature at every point. The torsional properties of diamond, gyroid, and primitive triply periodic minimal surface structures based on sheets were investigated using finite element analysis (FEA) by Skibar et al. (Contribution No. 6). It has been shown that primitive structures have a significantly higher shear modulus of 1500–2500 MPa depending on the conditions, compared to a shear modulus of 400–1000 MPa for diamond and gyroid structures. Similar relationships were obtained for the yield strength under shear, which was 44–73 MPa for primitive structures, while for diamond and gyroid structures it was 21–27 MPa. For all types of structures studied, relative density had a significant impact. As it increased, both the shear modulus and the yield strength in shear increased. The exception here are primitive structures with an increased unit cell (1.5 to 2 times larger). In this case, the effect of relative density was reversed: as relative density increased, the shear modulus and yield strength at shear decreased.

The deformations that occur during the curing of curved pultruded composites are mainly due to the asymmetric temperature distribution and the accumulated residual stresses generated during the forming process [26]. To address this issue, Wu et al. (Contribution No. 5) developed a simulation model based on FEA, incorporating a thermochemical model corrected for curvature and a strain-dependent constitutive equation. Taguchi orthogonal experiments combined with analysis of variance [27] were used to quantitatively assess the influence of process parameters on residual stresses. Among these parameters, the bending height was identified as a statistically significant factor. It was determined that the optimal process parameters are a bending height of 20 mm, a heating rate of 10 °C/min, a holding time of 16 s, and a pultrusion speed of 70 cm/min. Under these conditions, the residual stress was minimized to 1205.6 Pa, representing a reduction of 2.79% compared to the optimal group in the orthogonal experiments. The proposed simulation framework and optimized process parameters provide a solid theoretical basis and practical technical guidelines for the precise control of curing deformation in curved composite components manufactured by pultrusion [28].

The development of polymers capable of selectively interacting with metal-based nutrient cations, such as zinc and iron, is attracting increasing interest, particularly in the context of sustainable agriculture, where these materials can serve as controlled carriers or modulators of micronutrient availability in plants. Despite their importance, the mechanistic basis of these interactions remains poorly understood, particularly with regard to the structural modification of the polymer to enhance its affinity for these ions. In this context, theoretical approaches become essential tools for the rational design of such materials [29]. One such approach was presented by de Jesus Damante et al. (Contribution No. 7). They designed a model polymer receptor to investigate how the nature and position of electron-donating (–NH_2_) and electron-withdrawing (–NO_2_) substituents influence the recognition of Zn^2+^ and Fe^2+^ cations. Using a combination of density functional theory calculations, energy decomposition analysis using natural orbitals for chemical valence, electrostatic potential mapping, and the quantum theory of atoms in molecules method, the receptor-cation interactions were decomposed into electrostatic, Pauli repulsion [30], orbital, and dispersion components. The results showed that the stability of the complexes depends mainly on the orbital and electrostatic components, with Fe^2+^ forming the most stable complex (−393.57 kcal mol^−1^) compared to a similar Zn^2+^ complex (−288.80 kcal mol^−1^). Zn^2+^ complexes exhibited a wide range of regulation depending on the substituent pattern. It was found that electron-donating groups systematically enhance both the electrostatic and orbital components, while nitro substituents exhibit a pronounced positional effect, ranging from strong destabilization to significant stabilization of the Zn^2+^ binding. These results established molecular-level guidelines for the design of polymeric nanogels with tunable affinity and selectivity toward trace element cations in agricultural applications.

In response to the growing demand for sustainable manufacturing, 3D printing using recycled polyethylene terephthalate (RPET) offers an innovative waste-to-value conversion method. Although the application of RPET in additive manufacturing is attracting considerable interest from both academic and industrial sectors, its further development is hampered by significant challenges, in particular, high shrinkage during processing and poor mechanical properties of the final product. Some of these challenges are addressed in the paper by Irska et al. (Contribution No. 8), which focuses on the development of PCs based on RPET with favorable processing, thermal and mechanical properties. The regranulates were produced via a twin-screw extrusion [31] using polyethylene terephthalate flakes, multifunctional chain extenders and short glass fibers. The resulting PCs were characterized with respect to their processing, thermal, thermomechanical and mechanical properties. Modification with a chain extender containing epoxy groups effectively increased the molecular weight and improved the processability, while reinforcement with glass fibers enhanced the tensile properties of both injection-molded [32] and 3D-printed parts. The main advantage of the RPET systems developed in the paper is their delayed crystallization kinetics. The results obtained highlight the significant potential of the proposed PCs for extrusion-based additive manufacturing, as delayed crystallization promotes better interphase adhesion, lower volumetric shrinkage and excellent dimensional stability.

During rapid filling of large type IV hydrogen tanks made of PCs, significant temperature gradients occur due to both gas compression and the Joule-Thomson effect [33], which may compromise the integrity of the tank and adversely affect overall safety. In relation to this phenomenon, Kartal and Yoldaş (Contribution No. 9) proposed an innovative approach to active mixing involving tank rotation, which improves internal convective heat transfer and consequently minimizes temperature gradients. Computational Fluid Dynamics (CFD) modeling [34,35] was used to assess the effect of rotational speed on the temperature distribution inside the tested tank. The most notable reduction in peak temperatures, and thus the most effective cooling, was observed at a speed of 30 rad/s. This speed provides the best compromise between temperature uniformity, maximum temperature limitation and energy consumption in the case of rapid refueling in large composite hydrogen storage systems.

## 3. Conclusions

The contributions published in this Special Issue demonstrate the rapidly evolving role of advanced modeling, computational framework design, and experimental characterization in the development of PCs and PNs. Based on the collection of research presented, several key conclusions and overarching trends can be drawn:

1. Sustainable and eco-friendly materials: The use of natural fibers, agricultural waste, and bio-based additives (e.g., coffee grounds, wool fibers, and myo-inositol) in polymer matrices reflects a clear shift in the industry toward sustainable materials. These solutions offer competitive mechanical, thermal, and acoustic properties, as well as wear resistance, providing a viable and eco-friendly alternative to traditional synthetic materials in construction and insulation applications.
2. Multi-criteria optimization using hybrid modeling: Combining experimental design tools (such as RSM and Taguchi methods) with artificial intelligence techniques (ANNs and ML) has proven to be extremely effective. These hybrid approaches enable precise prediction of complex behaviors—such as wear resistance, mass-displacement relationships in LSs, and sound absorption—which significantly shortens design cycles.
3. Advanced numerical simulations of structural integrity: Advanced numerical modeling—including FEA and CFD—remains indispensable for capturing nonlinear phenomena. The research presented in this Special Issue demonstrates how simulations effectively help address technological challenges, such as controlling deformations during the curing of pultruded components, characterizing shear mechanisms in complex LSs, and mitigating critical temperature gradients during rapid refueling in composite hydrogen storage tanks.
4. Tailored multifunctionality and additive manufacturing: 3D printing using recycled polymer systems (e.g., RPET reinforced with short glass fibers) shows significant commercial and functional potential. Furthermore, theoretical quantum-chemical strategies and molecular dynamics simulations are successfully opening up new possibilities for the design of targeted polymer receptors with precisely tuned ionic selectivity for specialized applications in the field of bio-agriculture.

In summary, this Special Issue highlights that the combination of multiscale mathematical and numerical modeling, ML-based optimization, and eco-creative materials synthesis is essential for the development of the next generation of high-performance PCs and PNs.

## Abbreviations

The following abbreviations are used in this paper:

ANNs	Artificial Neural Networks
BCs	Biocomposites
CFD	Computational Fluid Dynamics
FEA	Finite element analysis
LSs	Lattice structures
ML	Machine learning
PCs	Polymer composites
PNs	Polymer nanocomposites
RPET	Recycled polyethylene terephthalate
RSM	Response Surface Methodology
SEM	Scanning Electron Microscopy
